# Spectroscopic Studies as a Toolbox for Biophysical and Chemical Characterization of Lipid-Based Nanotherapeutics

**DOI:** 10.3389/fchem.2018.00323

**Published:** 2018-07-31

**Authors:** Eduarda Fernandes, Telma B. Soares, Hugo Gonçalves, Marlene Lúcio

**Affiliations:** Department of Physics, Centre of Physics of University of Minho and Porto, University of Minho, Braga, Portugal

**Keywords:** nanotherapeutics' characterization, derivative spectroscopy, fluorescence quenching, steady-state anisotropy, HSA binding, fluorescence lifetime imaging microscopy

## Abstract

The goal of this study is to provide tools to minimize *trial-and-error* in the development of novel lipid-based nanotherapeutics, in favor of a rational design process. For this purpose, we present case-study examples of biophysical assays that help addressing issues of lipid-based nanotherapeutics' profiling and assist in the design of lipid nanocarriers for therapeutic usage. The assays presented are rooted in spectroscopic methods (steady-state and time-resolved fluorescence; UV-Vis derivative spectroscopy; fluorescence anisotropy and fluorescence lifetime image microscopy) and allow accessing physical-chemical interactions between drugs and lipid nanocarriers, as well as studying interactions between lipid-based nanotherapeutics and membranes and/or proteins, as this is a key factor in predicting their therapeutic and *off target* effects. Derivative spectroscopy revealed Naproxen's high distribution (Log*D* ≈ 3) in different lipid-based nanocarriers (micelles and unilamellar or multilamellar vesicles) confirming the adequacy of such systems for encapsulating this anti-inflammatory drug. Fluorescence quenching studies revealed that the anti-inflammatory drugs Acemetacin and Indomethacin can reach an inner location at the lipid nanocarrier while being anchored with its carboxylic moiety at the polar headgroup. The least observed quenching effect suggested that Tolmetin is probably located at the polar headgroup region of the lipid nanocarriers and this superficial location may translate in a fast drug release from the nanocarriers. Fluorescent anisotropy measurements indicated that the drugs deeply buried within the lipid nanocarrier where the ones that had a greater fluidizing effect which can also translate in a faster drug release. The drug binding strength to serum albumin was also compared for a free drug (Clonixin) or for the same drug after encapsulation in a lipid nanocarrier DSPC:DODAP (2:1). Under both conditions there is a strong binding to serum albumin, at one binding site, suggesting the need to produce a stealth nanosystem. Finally the cellular uptake of lipid nanocarriers loaded with Daunorubicin was investigated in cancer cells using fluorescence lifetime imaging microscopy. From the images obtained it was possible to conclude that even at short incubation times (15 min) there was a distribution of the drug in the cytoplasm, whereas for longer incubation periods (4 h) the drug has reached the nucleus.

## Introduction

In the latest century, Nanotechnology is one of the fastest increasing technological fields. Recent advances in the development of new materials with unique properties have provided exciting new opportunities in different areas, including environmental applications where nano adsorbents are gaining importance due to their remarkable capacities to uptake a wide variety of pollutants (Ali et al., 2015, 2016a,b,c,d,e, 2017). Simultaneously, Nanomedicine emerges as a continuously evolving area of research that uses Nanotechnology to produce nanotherapeutics aiming the progress in the prevention, therapy and diagnosis of a large spectrum of diseases (Duncan and Gaspar, 2011; Fornaguera and García-Celma, 2017). Nowadays, nanotherapeutics have become the promise to overcome many limitations faced by classical therapy, such as low bioavailability or poor pharmacokinetics, thus, having a major implication on public health improvement (Hafner et al., 2014; Fornaguera and García-Celma, 2017; Gao and Lowry, 2018). For example, nanotherapeutic's ability to provide targeted and/or triggered delivery of drugs, as well as an increase in drug solubility and decrease in toxicity, describes it as an essential application in treatment of numerous diseases. Additionally, nanotherapeutics promote the reduction of certain conventional drawbacks such as, poor compliance, non-specific delivery, toxicity related to higher doses, and early body clearance (Hafner et al., 2014).

Nowadays, despite the great optimism concerning Nanomedicine's potential impact, nanotherapeutics development is often made on basis of *trial-and-error* approaches with poor *in vitro* systematic characterization and without considering the rational design proposed by the pharmaceutical industries (Duncan and Gaspar, 2011; Hafner et al., 2014; Wacker, 2014). This leads to great investments in nanotherapeutics' development which fails to fulfill their expected therapeutic effects at a very late stage of the developmental process. In this regard, the EU Committee on strategy for nanotechnologies alerts for insufficient fundamental studies directed to understand nanotherapeutics' *in vivo* fate. Furthermore, Andrew D. Maynard and his co-authors (in a Nature publication) propose that the major challenges in nanotechnology for the next 15 years are the development of *in vitro* methods and models to predict the nanotherapeutics' *in vivo* behavior (Maynard et al., 2006). Moreover, the European Commission and the European Technology Platform for Nanomedicine suggests that a major challenge in nanotherapeutics' development is the successful translation of research from academia to production lines with efficacy, safety, and quality controls to improve the *quality-by-design* approach (Duncan and Gaspar, 2011; Hafner et al., 2014). Failures in research to market translation justify that only a small number of nanotherapeutics have been approved and commercialized. A comprehensive analysis of the worldwide state of investigational and approved nanomedicine products as of January 2012 has identified a still very short list of marketed nanotherapeutics (Etheridge et al., 2013). From the 21 marketed approved nanocarriers for drug delivery 15 are liposomes or lipid based nanocarriers (AmBisome®, DaunoXome®, DepoCyt®, DepoDur®, Doxil®, Inflexal® V, Marqibo®, Mepact®, Myocet®, Visudyne®, Abelcet®, Amphotec®, Fungizone®, Diprivan®, Estrasorb®) whereas 3 are protein drug conjugates (Eligard®, Genexol®, Opaxio®) and other 3 are polymer based nanoformulations (Abraxane®, Kadcyla®, Ontak®) (Weissig et al., 2014).

From a general standpoint lipid nanocarriers are better accepted by pharmaceutical companies for therapeutic purposes, since its lipid components are generally recognized as safe (GRAS). Furthermore, lipid nanocarriers are biodegradable, and for that reason considered to not accumulate in the body and regarded as possibly risk-free (Qi et al., 2017).

Therefore, this study aims to provide a bio-inspired approach for design and develop lipid-based nanotherapeutics (NT) based in “*in vitro*” biophysical techniques. For this purpose, we present case-study examples of assays that address issues of NT profiling, as well as, assist in the design of lipid nanocarriers for therapeutic purposes.

**Figure 1:**
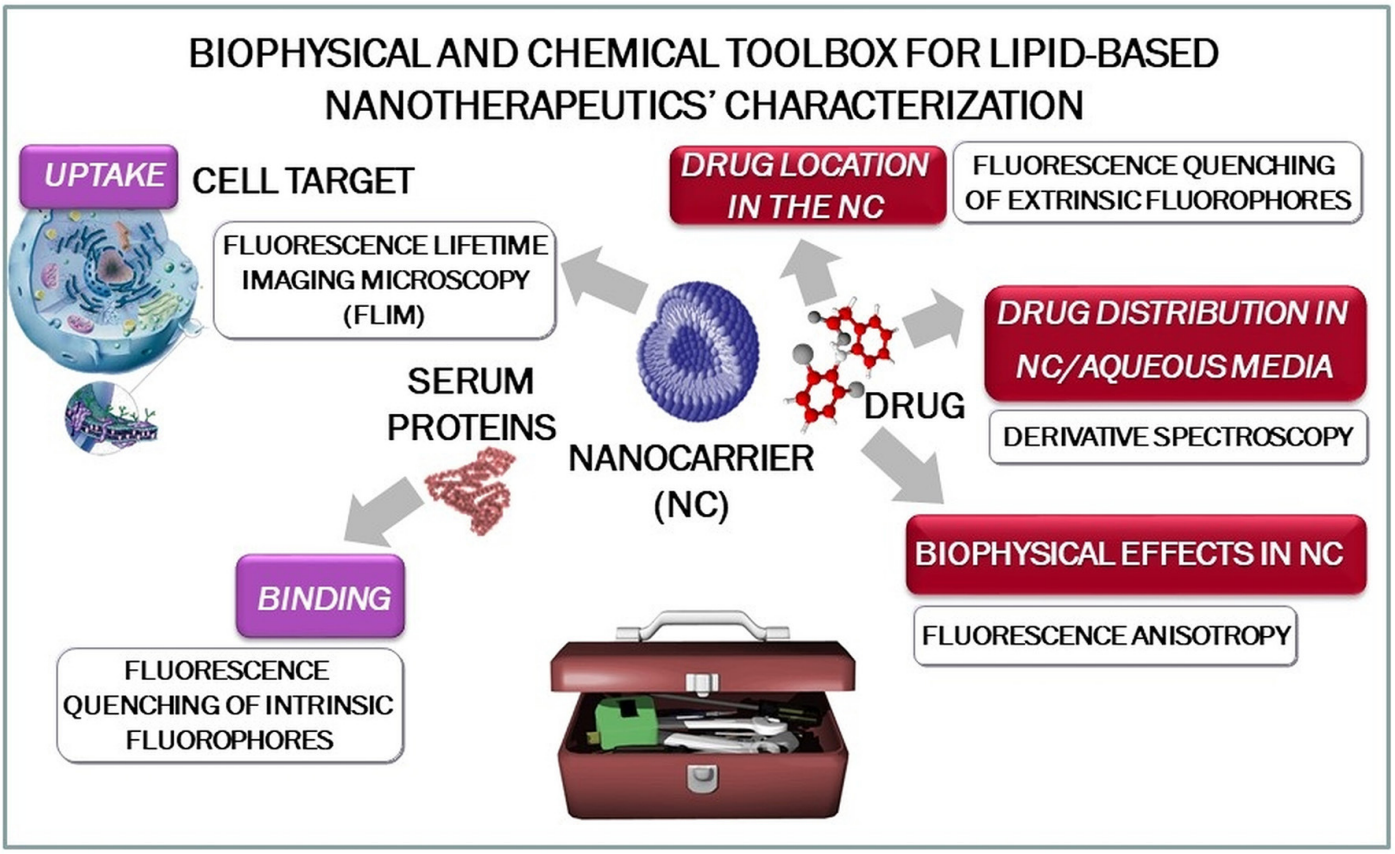
Schematic representation of the biophysical and chemical toolbox for lipid-based nanotherapeutics' characterization.

To simplify the practical use of this work, it will be presented as a toolbox for biophysical-chemical characterization of NT that can be applied to several lipid formulations (e.g., lipid vesicles, lipid micelles, cubosomes, solid lipid nanoparticles, nanostructured lipid carriers, ethosomes, niosomes, etc.) and therapeutic agents (Figure 1).

The examples presented are divided into two major topics: (i) *pre-formulation studies*, focusing on the interactions between the drug and the nanocarrier, and (ii) *performance studies* with a focus on the interactions between NT and biological structures. In the section of pre-formulation studies, we present strategies to analyse drug distribution, drug location and drug effect on NT's biophysical properties. Drug distribution coefficient (Log*D*) between the lipid phases of the NT and the aqueous phase is studied by derivative spectroscopy, leading to the determination of drug encapsulation. Drug location in NT is inferred by fluorescence quenching, where a decrease on fluorescence intensity of extrinsic probes was measured to indicate drug distribution and possible orientation within NT. Furthermore, drug effect on biophysical properties of NT is studied using fluorescence anisotropy, because for instance a great increase of the fluidity of the NT lipid system can turn it into a very leaky and not too stable nanocarrier for the drug. In the latter section, strategies are presented focusing on analyzing: NT interaction with plasma proteins by measuring fluorescence quenching of intrinsic probes, as this is paramount to assure that the nanocarrier will reach target tissues and will not have an exceeding circulation time and finally, cellular uptake of drug will be analyzed by fluorescence-lifetime imaging microscopy (FLIM).

## Materials and methods

### Reagents

The studied drugs (Acemetacin, Indomethacin, Tolmetin, Naproxen, Clonixin and Daunorubicin), 12-anthroyloxi-stearic acid (12-AS) probe and Human serum albumin (HSA) were obtained from Sigma-Aldrich Química, S.L. (Sintra, Portugal). The other *n*-anthroyloxi-stearic acid probes designated as *n*-AS probes (2-AS, 6-AS and 9-AS) were supplied by Molecular Probes (Invitrogen Corporation, Carlsbad, California, USA). All the reagents were used as supplied without further purification. All other chemicals were purchased from Sigma-Aldrich Química, S.L. (Sintra, Portugal) with p.a. or similar quality. Phospholipids: Egg Phosphatidylcholine (EPC), Hexadecylphosphocholine (HDPC); and 1,2-dioleoyl-3-dimethylammonium-propane (DODAP were obtained from Avanti Polar Lipids (INstruchemie, Netherlands). All lipids were dissolved in Chloroform pro analysis (p.a) from Sigma-Aldrich Química, S.L. (Sintra, Portugal) to obtain stock solutions of 20 mM.

### Preparation of lipid nanocarriers

Lipid nanocarriers with different structures, compositions and surfaces charges and sizes were prepared for their wide use as drug nanocarriers: large unilamellar vesicles of 100 nm (LUVs), multilamellar vesicles (MLVs), and micelles.

EPC liposomes were prepared in Hepes buffer (pH = 7.4; *I* = 0.1 M adjusted with NaCl) by the lipid film hydration method, described in Lasic (1993) and then MLVs were used without manipulation while LUVs were obtained after successive extrusions of the MLVs suspension through Nuclepore Track-Etched polycarbonate membrane filters with different pore diameters (400, 200, and 100 nm) in a Lipex extruder to obtain LUVs. The HDPC micellar suspensions were prepared with bi-deionized water (conductivity < 0.1 μS cm^−1^) and Hepes buffer (10 mM, pH = 7.4; *I* = 0.1 M with NaCl).

In the case of LogD determination studies, for each lipid nanocarrier system model used (HDPC micelles and MLVs or LUVs of EPC), two groups of suspensions were prepared. The first one—the samples—were prepared containing a fixed concentration of Naproxen and increasing concentrations of lipid suspension. The second group—the references—were identically prepared without addition of Naproxen. All suspensions were subsequently incubated during 30 min at 25.0°C.

To obtain labeled lipid model systems, ethanolic stock solutions of fluorescent probes were added to the pre-formed EPC liposomes suspensions in an amount such that the lipid: probe ratio is always >100:1 (m:m), to prevent structural changes of the nanocarrier system. The addition of the probe was done under gentle shaking, followed by 30 min incubation at room temperature and sheltered from light promoting the total incorporation of the probe into the lipid bilayer (New, 1990; Lasic, 1993). Increasing concentrations of NSAIDs (Tolmetin, Indomethacin and Acemetacin) prepared in Hepes buffer were added to the lipid suspensions labeled with 2, 6, 9 or 12-AS probes (500 μM) to obtain final drug concentration of 0–600 μM. After mild shaking, the suspensions were protected from light and incubated at room temperature for 2 h.

LUVs of DSPC:DODAP (2:1) loaded with Clonixin or loaded with Daunorubicin were produced from a direct mixing in an organic solvent mixture (chloroform: ethanol 8:2) of an appropriate amount of DSPC, DODAP and drug (Clonixin: 1.52 × 10^−3^ M or Daunorubicin: 0.5 μM) by the lipid film hydration method followed by extrusion as previously described for EPC liposomes.

### Derivative spectroscopy

The absorption spectra of samples and references were plotted in the 250–500 nm range, on a Perkin-Elmer Lambda 45 UV-Vis spectrophotometer equipped with a thermostated cell holder at 25.0 ± 0.1°C. Derivative spectra were made from absorption spectra after subtracting the absorption of the references and using an Excel® based routine previously published (Magalhães et al., 2010).

The fluorescence emission spectra of samples and references were obtained in the 300–500 nm range, at a scanning rate of 50 nm.min^−1^, during 10 s integration time on a Perkin-Elmer LS-50B spectrofluorimeter equipped with a thermostated cell holder at 25.0 ± 0.1°C and λ_excitation_ = 272 nm. Three independent experiments were carried out. Derivative spectra were made from fluorescence emission spectra using Microcal Origin 9.0® software further used for data treatment.

### Steady-state spectrofluorimetry using extrinsic probes

The fluorescence excitation and emission spectra of nanocarriers labeled with *n*-AS probes were plotted on Perkin-Elmer LS-50B spectrofluorimeter using quartz cells of 10 mm optical path themostated at 25.0 ± 0.1°C. The excitation wavelength was defined to 381 nm and emission wavelength were set to 452 nm (2-AS), 446 nm (6 and 9-AS) and 444 nm (12-AS) with excitation and emission slits between 3 and 6 nm. For correction of the inner filter effect (Lakowicz, 2014), absorbance spectra of analyzed suspensions were obtained using a Perkin-Elmer Lambda 45 UV-Vis with 1 cm quartz cell thermostated at 25.0 ± 0.1°C, with a scanning speed of 400 nm.min^−1^. Steady-state anisotropy determination was performed in Perkin-Elmer LS-50B, with quartz cell with an optical path of 10 mm, thermostated at 25.0 ± 0.1°C and with automatic insertion of vertical and horizontal polarizers. Temperature value was selected to be well over the transition temperature of the lipid components to ensure that the lipid nanocarrier is in the fluid phase and thus warrant the free rotation of the probe, i.e., *r*_∞_≈ 0) (Vincent et al., 1982). The measurements were performed with an integration time of 50 s, after polarizer placement and with excitation and emission slits between 3 and 6 nm. The excitation wavelength was defined to 381 and the emission wavelength was set up considering the probe: 452 nm (2-AS); 446 nm (6-AS and 9-AS), and 444 nm (12-AS). Three independent experiments were carried out.

### Steady-state spectrofluorimetry using intrinsic probes

For HSA-clonixin binding measurements, increasing drug amounts were added to solutions of HSA at a fixed concentration (concentration of 6 × 10^−4^ M equivalent to HSA blood plasma concentration (Ma and Hadzija, 2013) freshly prepared in HEPES buffer (pH 7.4; *I* = 0.1 M). For binding measurements between HSA and liposomes loaded with Clonixin, different volumes of LUVs of DSPC:DODAP (2:1) loaded with 15% of Clonixin were added to the same fixed concentration of HSA to achieve final lipid concentrations of 1 × 10^−5^, 5 × 10^−5^, 1 × 10^−4^, 5 × 10^−4^, 1 × 10^−3^, and 1.5 × 10^−3^ M.

Fluorescence quenching measurements of HSA intrinsic emission were carried out with a spectrofluorometer, LS-50B (Perkin Elmer, Waltham, MA) using quartz cells of 10 mm optical path. The excitation wavelength was defined to 280 nm and emission spectra was recorded between 300 and 450 nm. The temperature was kept constant and equal to physiological temperature 37 ± 0.5°C. The excitation and emission slit widths were set to 2 nm. Three independent experiments were carried out.

### Fluorescence life-time imaging microscopy (FLIM)

For FLIM analysis, Human cervical carcinoma (Hela) cells were seeded into sterilized 3 cm glass bottom dishes (Mat-Tek) (100,000 cells/well). After 24 h seeding, the medium was replaced with fresh medium containing LUVs of DSPC:DODAP (2:1) loaded with Daunorubicin (0.5 μM equivalent drug concentration), and cells were incubated at 37°C for 15 min and 4 h. The fluorescence lifetime distribution was measured by a homemade FLIM setup (Bernardo et al., 2014) based in a femtosecond laser Ti:sapphire with a natural frequency of 800 nm and 100 fs pulse duration as a radiation source. The decay profiles were recorded using the double of the laser frequency as an excitation source (λexc = 400 nm) and the emission was collected at λemi = 592 nm corresponding to the maximum of the fluorescent band of the drug (Than Htun, 2009).

## Results

### Pre-formulation studies

#### Distribution coefficient studies to evaluate interaction between drug and lipid nanocarriers

To understand the numerous mechanisms related to a drug loaded in a lipid colloidal based nanocarrier, it is important to study the interactions between the drug and the lipid components of the nanocarrier or between the drug and a mimetic model system of the nanocarrier used for drug encapsulation. One of the most important parameters to evaluate these interactions is the drug distribution between lipid/aqueous phases, which allows evaluating if the drug is more distributed in the hydrophobic or hydrophilic microenvironment of the nanocarrier. Considering the type of nanocarrier designed there are several models that may be used to determine drug distribution coefficient (Log*D*): micelles and lipid vesicles (LUVs and MLVs). The Log*D* of Naproxen, a nonsteroidal anti-inflammatory drug (NSAID), in different biphasic lipid/aqueous systems will be presented as an example of this pre-formulation study.

**Figure 2:**
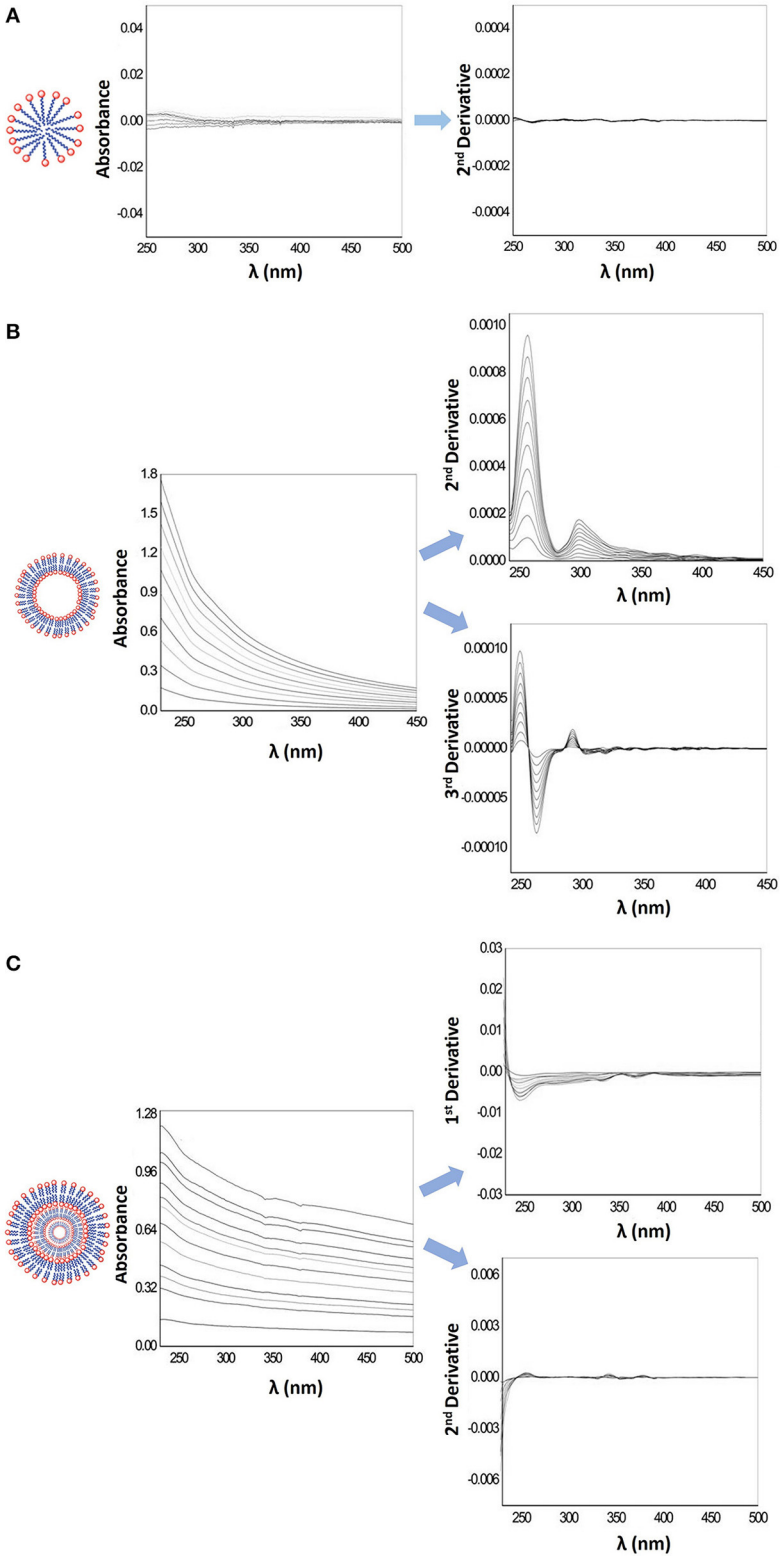
**(A)** Representative scheme of a micelle, its absorption spectra and second derivative spectra obtained for increasing HDPC concentrations (100–900 μM) in Hepes buffer pH = 7.4 and *I* = 0.1 M. **(B)** Representative scheme of LUVs, its absorption spectra and second and third derivative spectra obtained for increasing concentrations liposomes (100–1,000 μM) in Hepes buffer pH = 7.4 and *I* = 0.1 M. **(C)** Representative scheme of MLVs, its absorption spectra and first and second derivative spectra obtained for increasing concentrations of liposomes (40–350 μM) in Hepes buffer pH = 7.4 and *I* = 0.1 M.

In Figure 2, it is possible to observe that the scattering due to the lipid vesicles is different according to the model system used. MLVs (Figure 2C) cause scattering of incident light, so the application of derivative spectroscopy is required. The first derivative of the spectra partly eliminates the interference caused by the scattering of light, while the second derivative eliminates completely the light scattering interferences, allowing a better distinction of characteristic spectral details of a drug/lipid interaction. LUVs suspensions (Figure 2B), although more translucent than MLVs suspensions can also cause incident light scattering in absorption spectra. Derivative spectroscopy allows partial elimination of this type of interference but not its total suppression, as can be seen in Figure 2. This is due to a marked Rayleigh dispersion which leads to a noticeable increase in light dispersion as it progresses to smaller wavelengths (Braun, 1987; Gorog, 2018). The spectral interference caused by HDPC micelles (Figure 2A) is very small, thus, the Log*D* calculation can be performed either directly from the absorption spectra or from the derivative spectra that leads to the complete elimination of the light scattering interference. The mathematical treatment of all spectra was performed using a previously developed routine, *Kp calculator* (Magalhães et al., 2010).

**Figure 3:**
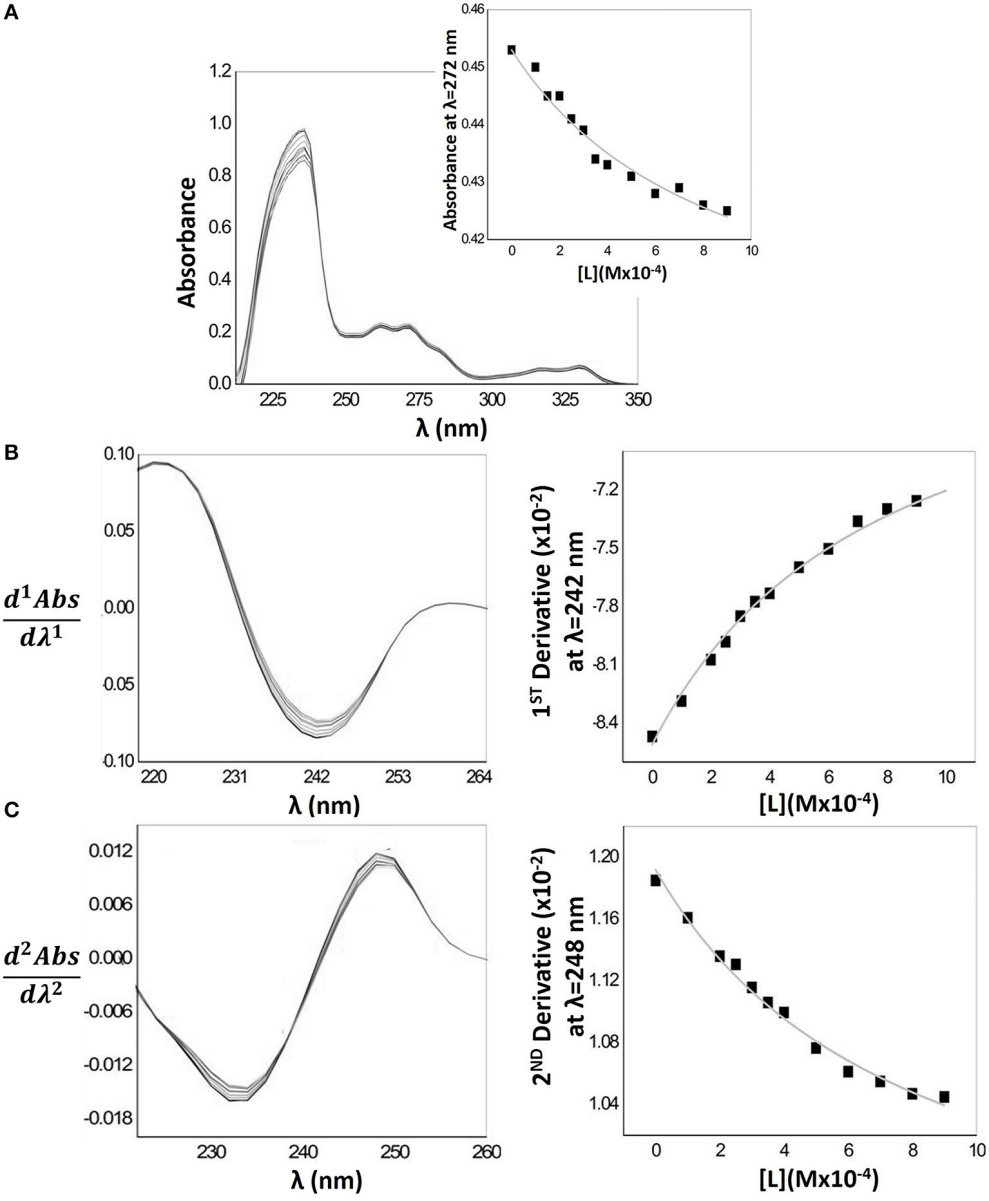
Schematic representation of mathematical treatment (fittings according Equations 1, 2) applied to Naproxen experimental absorption spectra with increasing concentrations of HDPC micelles (0–900 μM) in Hepes buffer pH = 7.4 and *I* = 0.1 M. **(A)** Naproxen absorption spectra including as an inset the plot of drug absorbance values at λ_max_ = 272 as a function of lipid concentration and the respective fitting by non-linear regression method. **(B)** First derivative of the Naproxen absorption spectra and the correspondent fitting at λ_max_ = 242 nm. **(C)** Second derivative of the Naproxen absorption spectra and the correspondent fitting at λ_max_ = 248 nm.

Considering the facts presented before, Log*D*_micelle/aqueous_ of Naproxen was estimated directly from the λ_max_ of absorption spectra (Figure 3A). In the figure, an example of the non-linear regression is represented (Abs vs. [L]) obtained by adjusting Equation 1 to the experimental data: absorbance (Abs) at λ_max_ = 272 nm obtained for increasing concentrations of HDPC micelles ([L]).

In the fitting expression, *T, a* and *l* subscripts stand respectively for total, aqueous and lipid and *D* is the distribution coefficient from which Log*D* is calculated.

(1) $$\begin{array}{r} Abs_{T}=Abs_{a}+\frac{\left(Abs_{l}-Abs_{a}\right)D\left[L\right]V_{l}}{1+D\left[L\right]V_{l}} \end{array}$$

The same fitting was applied to the other absorption λ_má*x*_ (262 and 330 nm) and Log*D*-values are represented in Table 1.

**Table 1:** Values of lipid/aqueous distribution coefficient (D and LogD) of Naproxen obtained from the absorbance spectra and from the derivative of the absorbance spectra.

**Log*****D*** **of Naproxen in a lipid/aqueous system determined by spectrophotometry and derivative spectrophotometry**						
	***D*****-values calculated from the absorbance spectra (Equation 1)**			***D*****-values calculated from the derivative spectra (Equation 2)**		**Average Log** ***D***^a^
				**1st Derivative**	**2nd Derivative**	
HDPC Micelles	λ = **262 nm**	λ = **272 nm**	λ = **330 nm**	λ = **242 nm**	λ = **248 nm**	
	1,091 ± 389	1,131 ± 304	1,233 ± 78	1,279 ± 79	1,266 ± 73	3.08 ± 0.07
EPC MLVs				λ = **244 nm**	λ = **250 nm**	
		ND		1,644 ± 77	1,441 ± 40	3.30 ± 0.03^***;^^ns^
**Log*****D*** **of Naproxen in a lipid/aqueous system determined by derivative spectrofluorimetry**				
	***D*** **values calculated from the fluorescence emission spectra (Equation 3)**			***D*** **values calculated from the 1st derivative spectra (Equation 4)**		**Average Log** ***D***^a^
EPC MLVs	λ = **359 nm**	λ = **344 nm**	λ = **376 nm**	
	3,244 ± 150	1,809 ± 194	1,812 ± 190	3.25 ± 0.04^ns^
EPC LUVs	λ = **359 nm**	λ = **344 nm**	λ = **376 nm**	
	3,950 ± 450	2,237 ± 524	2,544 ± 421	3.38 ± 0.09^****^;^ns^

a *The value corresponds to the mean and respective standard deviation obtained for all wavelengths of at least three independent assays. Comparisons were performed using a Krauskal-Wallis test followed by Dunn's Post-hoc test, for the following paired LogD values obtained for MLVs and LUVs vs. LogD values obtained for micelles; LogD values obtained for MLVs vs. LogD values obtained for LUVs*.

**** *P < 0.0001*,

*** *P < 0.001*;

ns *, not significant; ND, not determined*.

The Log*D* determination by derivative spectrophotometry is based on a similar equation where instead of values taken at the λ_max_ of the absorbance spectra, values are taken at the peaks of the correspondent *n* order derivative spectra where the light scattering interference of the nanocarrier has been eliminated (Ferreira et al., 2003, 2005a,b; Lúcio et al., 2006):

(2) $$\begin{array}{r} \frac{d^{n}Abs_{T}}{d\lambda^{n}}=\frac{d^{n}Abs_{a}}{d\lambda^{n}}+\frac{\left(\frac{d^{n}Abs_{l}}{d\lambda^{n}}-\frac{d^{n}Abs_{a}}{d\lambda^{n}}\right)D\left[L\right]V_{l}}{1+D\left[L\right]V_{l}} \end{array}$$

In the aforementioned equations, the lipid concentration [*L*] expressed in molL^−1^ is multiplied by the calculated volume of the lipid nanocarrier in Lmol^−1^ (*V*_*l*_) to obtain a dimensionless value of Log*D* that can be compared with others Log*D* values described in the literature for other systems (Lúcio et al., 2006).

Although, in the case of drugs distributed in the micelle/aqueous phase system, it is possible to calculate Log*D* from the absorption spectra, the derivative spectroscopy can also be applied to increase the resolution of poorly differentiated bands (Kitamura and Imayoshi, 1992; Kitamura et al., 1995; Omran et al., 2001). Theoretically, Log*D* values can be calculated from *d*^*n*^*Abs*_*T*_*/d*λ^*n*^ at any wavelength. However, to increase signal/noise ratio, the *d*^*n*^*Abs*_*T*_*/d*λ^*n*^ values for heterogeneous samples should be obtained for λ_max_ or λ_min_ values of the derivative spectra (peaks of the derivatives). Log*D* values obtained for different peaks of the derivatives (1^st^ derivative, λ = 242 nm and 2^nd^ derivative, λ = 248 nm)—represented in Table 1—were shown to be concordant confirming the total elimination of the light scattering caused by micelles.

Figure 3 also represents the first (Figure 3B) and second (Figure 3C) derivative spectra where it is possible to observe a shift of λ_max_ characteristic of a drug distribution into environments of lower polarity (distribution of Naproxen into the lipid phase) (Kitamura et al., 1995), with a decrease in the intensity of the bands as the lipid concentration increases. Isosbestic points are also observed indicating the existence of a balance between two drug forms (in interaction with the lipid medium and free in aqueous medium) and the eradication of the light scattering interference (Saakov et al., 2015).

From Table 1 it is worth noting that results obtained from the UV/Vis spectra and derivative spectra give very similar values of Log*D*, confirming that the insignificant light scattering interference of micelles is easily eliminated by derivation of the absorption spectra. Thus, the average Log*D* value obtained for Naproxen distribution in the micelle/aqueous phase is 3.08 ± 0.07.

**Figure 4:**
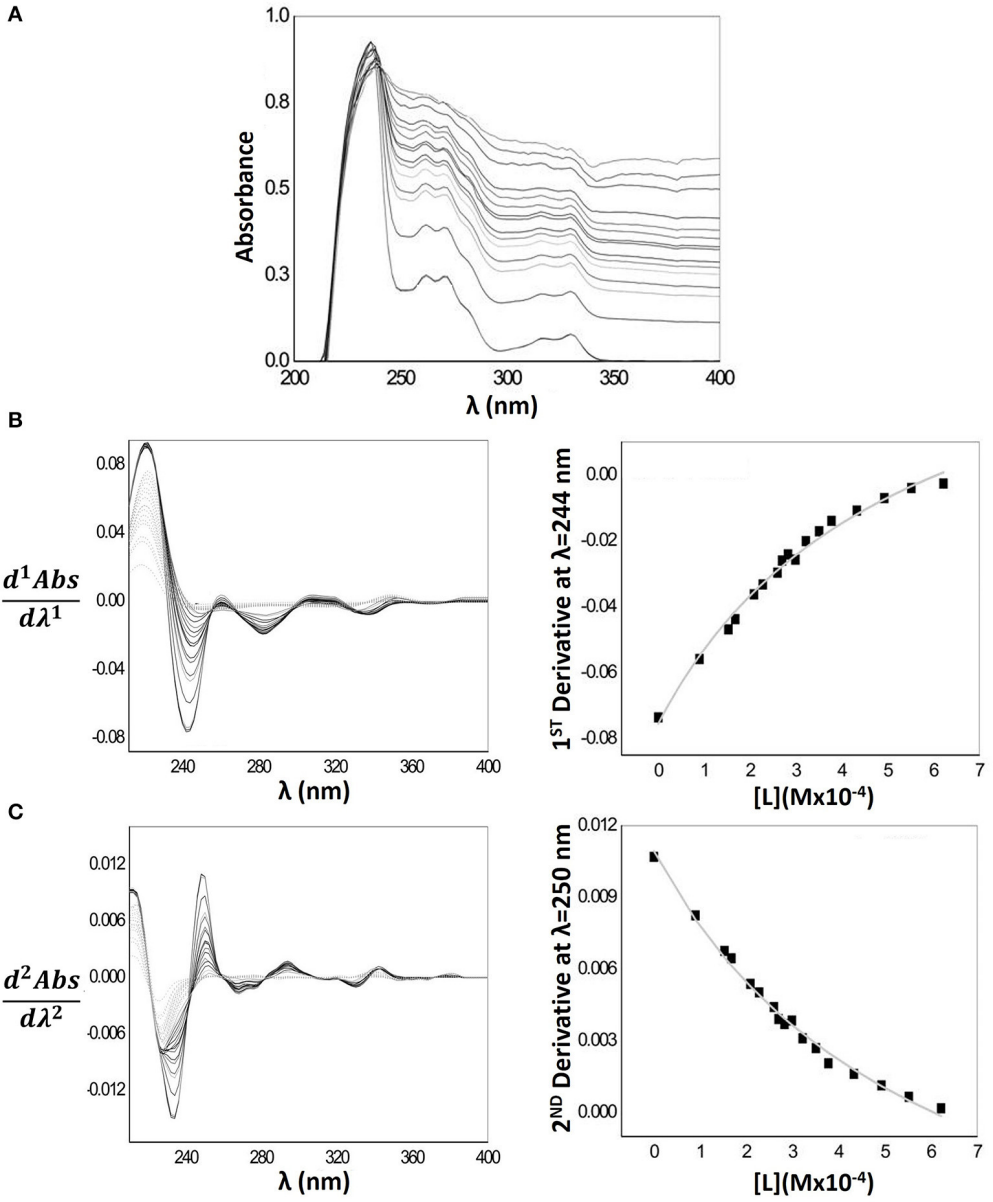
**(A)** Naproxen absorption spectra with increasing concentrations of MLVs of EPC (0–622 μM) in Hepes buffer pH = 7.4 and *I* = 0.1 M **(B)** First derivative spectra (left) and correspondent nonlinear fitting (right) of derivative absorbance values at λ_max_ = 244 nm as a function of lipid concentration [L]. **(C)** Second derivative spectra (left) and correspondent nonlinear fitting (right) of derivative absorbance values at λ_max_ = 250 nm as a function of lipid concentration [L].

Figure 4A represents the Naproxen absorption spectra when encapsulated in MLVs nanocarriers. The baseline of the absorption spectra increases greatly with increasing MLVs concentration, indicating a light scattering effect. Hence, for a proper quantification of Naproxen Log*D* the first and second derivatives of the spectra were required. In Figure 4B, it is possible to observe that the interference of the light scattering effect is minimal for λ > 235 nm values. Indeed, from these values of λ, the references of 1^st^ derivative absorption spectra (dashed light gray lines) have a signal close to zero, making possible the calculation of Naproxen Log*D* at λ = 244 nm. The same trend can be observed in the second derivative spectra (Figure 4C); however, the light scattering is minimal for λ > 240 nm, being possible to determine Naproxen Log*D* at λ = 250 nm. The fittings of Equation 2 to the experimental data, through a non-linear regression, that were used to obtain the *D* and Log*D* values presented in Table 1, are also represented in the same figure.

The Log*D* value of a drug can also be determined by spectrofluorimetry, if the drug is a fluorescent compound, and if there is a variation in a fluorescence parameter (e.g., fluorescence emission intensity, λ_max_ of the emission) due to its distribution in lipid/aqueous phases (Santos and Castanho, 1996; Pereira-Leite et al., 2013). The fluorescence emission intensity, *I*, can be used to calculate the distribution *D*, and the correspondent Log*D* of a fluorescent drug between the lipid and aqueous phases, through Equation 3:

(3) $$\begin{array}{r} I_{T}=I_{a}+\frac{\left(I_{l}-I_{a}\right)D\left[L\right]V_{l}}{1+D\left[L\right]V_{l}} \end{array}$$

This equation is similar to that presented for the determination of *D* by spectrophotometry (Equation 1). However, instead of absorbance, it is measured the fluorescence emission intensity (*I*_*T*_) of the drug distributed in lipid nanocarriers accounting for the fluorescence emission intensity contributions of drug distributed in the lipid (*I*_*l*_) and aqueous (*I*_*a*_) phases which can be related to *D* and then Log*D* values (Santos and Castanho, 1996; Santos et al., 1998).

**Figure 5:**
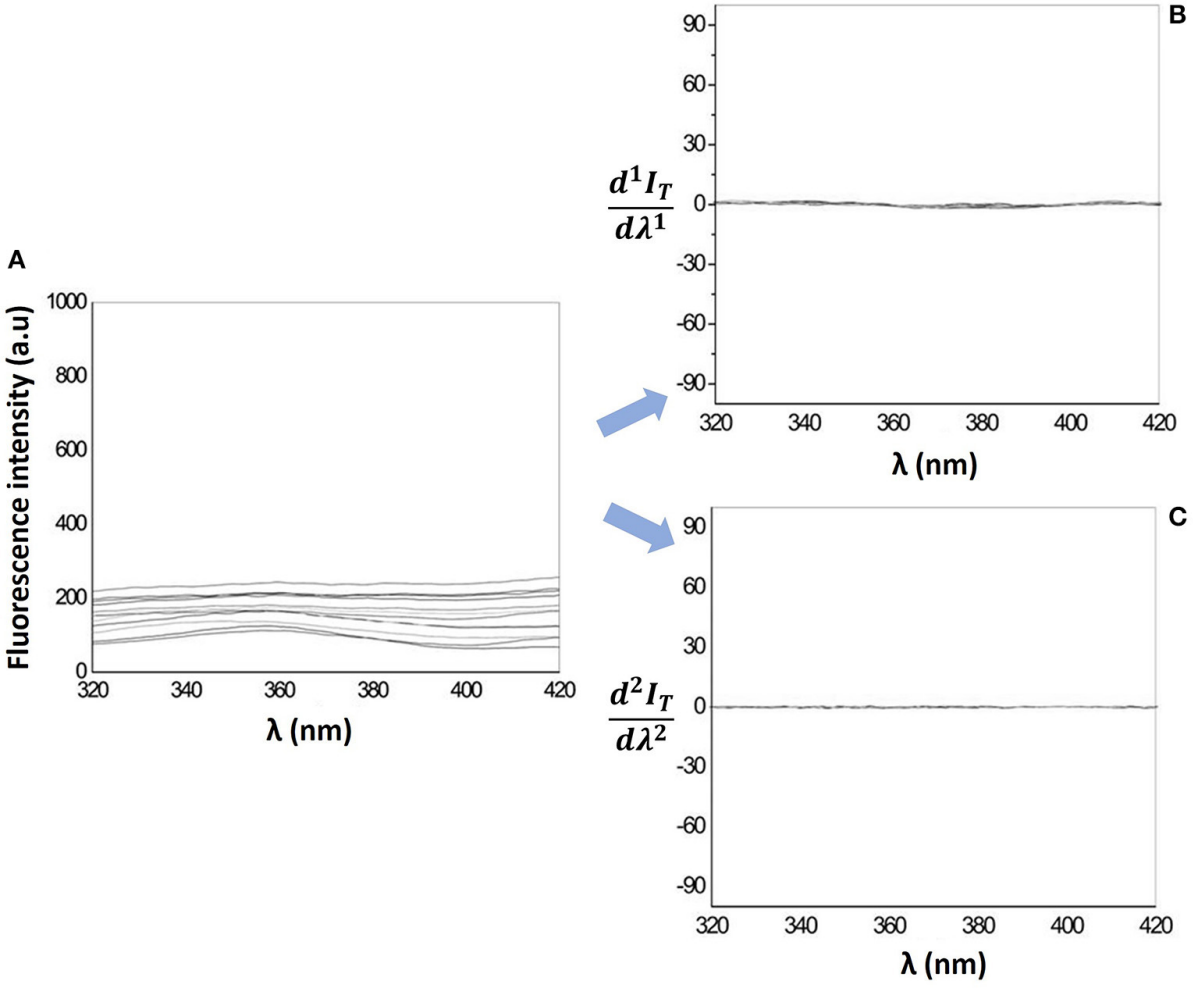
**(A)** Fluorescence emission spectra and its first derivative spectra **(B)** and second derivative spectra **(C)** for increasing concentrations of MLVs of EPC (25–300 μM) in Hepes buffer, pH = 7.4 and *I* = 0.1 M.

The determination of drug Log*D* in lipid/aqueous phase by fluorescence methods is more advantageous over UV-Vis spectrophotometric methods, since the light scattering caused by the lipid media is reduced (Figure 5A) and can be totally eliminated with the 1^st^ and 2^nd^ derivative of the fluorescence emission spectra (Figures 5B,C).

The Log*D* determination by derivative spectrofluorimetry is based on a similar equation where instead of values taken at the λ_max_ of the fluorescence emission spectra, the values are taken at the peaks of first derivative spectra where the nanocarrier light scattering interference has been eliminated (Ferreira et al., 2003, 2005a,b; Lucio et al., 2004):

(4) $$\begin{array}{r} \frac{d^{1}I_{T}}{d\lambda^{1}}=\frac{d^{1}I_{a}}{d\lambda^{1}}+\frac{\left(\frac{d^{1}I_{l}}{d\lambda^{1}}-\frac{d^{1}I_{a}}{d\lambda^{1}}\right)D\left[L\right]V_{l}}{1+D\left[L\right]V_{l}} \end{array}$$

Naproxen is a fluorescent drug with λ_max_ of excitation at 262, 272 and 330 nm. The Log*D* of Naproxen in a LUVs/aqueous media cannot be determined by derivative spectrophotometry since λ_max_ of the derivative spectra (244 and 250 nm) correspond to values of λ where spectral interference of LUVs is very high (Figure 2B). Therefore, the Log*D* of Naproxen in a LUVs/aqueous media was determined by spectrofluorimetry and derivative spectrofluorimetry. For comparison with the results obtained by derivative spectrophotometry, the Log*D* of Naproxen in a MLVs/aqueous media was also determined by derivative spectrofluorimetry (Table 1).

**Figure 6:**
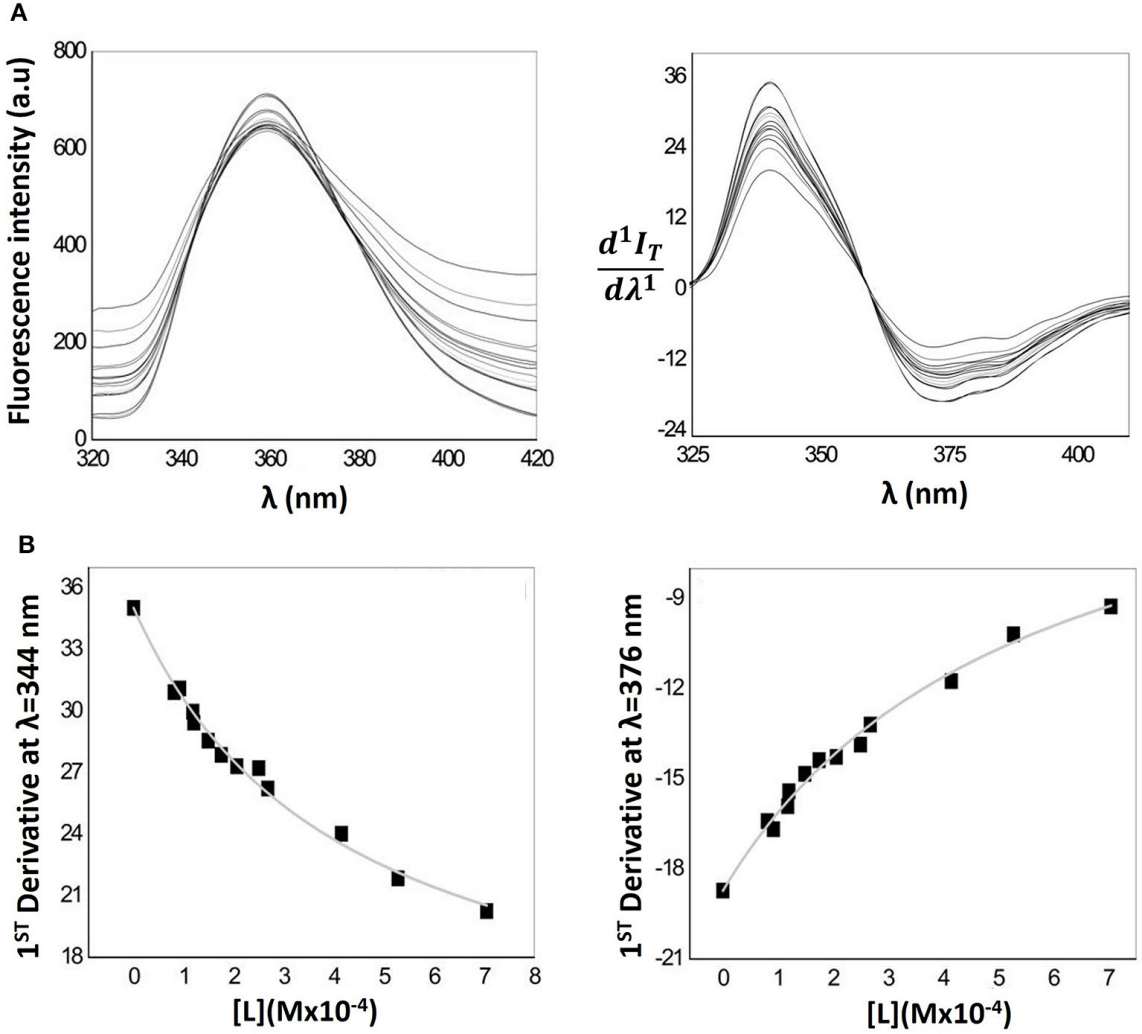
**(A)** Fluorescence emission spectra (left) of MLVs of EPC (25–300 μM) containing a fixed Naproxen concentration (0.4 μM) in Hepes buffer pH = 7.4 and *I* = 0.1 M and respective first derivative of Naproxen fluorescence emission spectra (right). **(B)** Correspondent nonlinear fitting (Equation 4) of derivative fluorescence emission values at λ_max_ = 344 nm (left) and at λ_min_ = 376 nm (right) as a function of lipid concentration.

The fluorescence emission spectra obtained for lipid nanocarriers containing Naproxen (Figure 6A left) shows a maximum fluorescence intensity at λ = 359 nm. Because of the light scattering caused by the lipid phase, the emission spectrum of Naproxen in the presence of MLVs requires the application of the derivative method, applying Equation 4 to the emission spectra. The first derivative spectra obtained are shown in Figure 6A (right). In this latest figure a well-defined isosbestic point can be observed indicating elimination of the effects related to residual light scattering. By derivative spectrofluorimetry, the calculation of *D* and then Log*D* of Naproxen was performed by fitting Equation 4 to the values of 1^st^ order derivative peaks (at λ = 344 nm and λ = 376 nm) as a function of lipid concentration (*d*^1^*I*_*T*_*/d*λ^1^ vs. [L]) through a non-linear regression (Figure 6B left and right). Although the spectral interference caused by the emission of the lipid phase is small until high concentrations of lipid are reached, its complete elimination occurs by application of the 1^st^ derivative, validating the derivative method for Log*D* determination by spectrofluorimetry.

The average Log*D* value obtained for Naproxen distribution in the MLVs/aqueous phase is 3.25 ± 0.04 and, in the LUVs/aqueous phase is 3.38 ± 0.09.

#### Studies to predict drug location in lipid nanocarrier

After quantifying the drug distribution between the lipid and aqueous phases of the nanocarrier system, it is important to assess where the drug will be most likely located within the lipid phase of the nanocarrier.

For this purpose, it is possible to label the lipid nanocarrier system with fluorescent extrinsic fluorophores, such as *n*-AS probes. *n*-AS probes are made of stearic acid with an anthracene fluorescent group located at different points of the acyl chain (e.g., 2, 6, 9, and 12). When incorporated in a lipid nanocarrier, their position will be well-defined and they will work as molecular rulers capable of labeling different depths (Lakowicz, 2014). In this regard, labeled nanocarriers (MLVs of EPC) were used to incorporate three NSAIDs: Tolmetin, Indomethacin and Acemetacin as an example of the application of these studies.

For all three NSAIDs tested a decrease in the intensities of fluorescence emitted by the fluorophores incorporated in MLVs can be observed. Additionally, the fluorescence quenching effect observed is concentration dependent.

**Figure 7:**
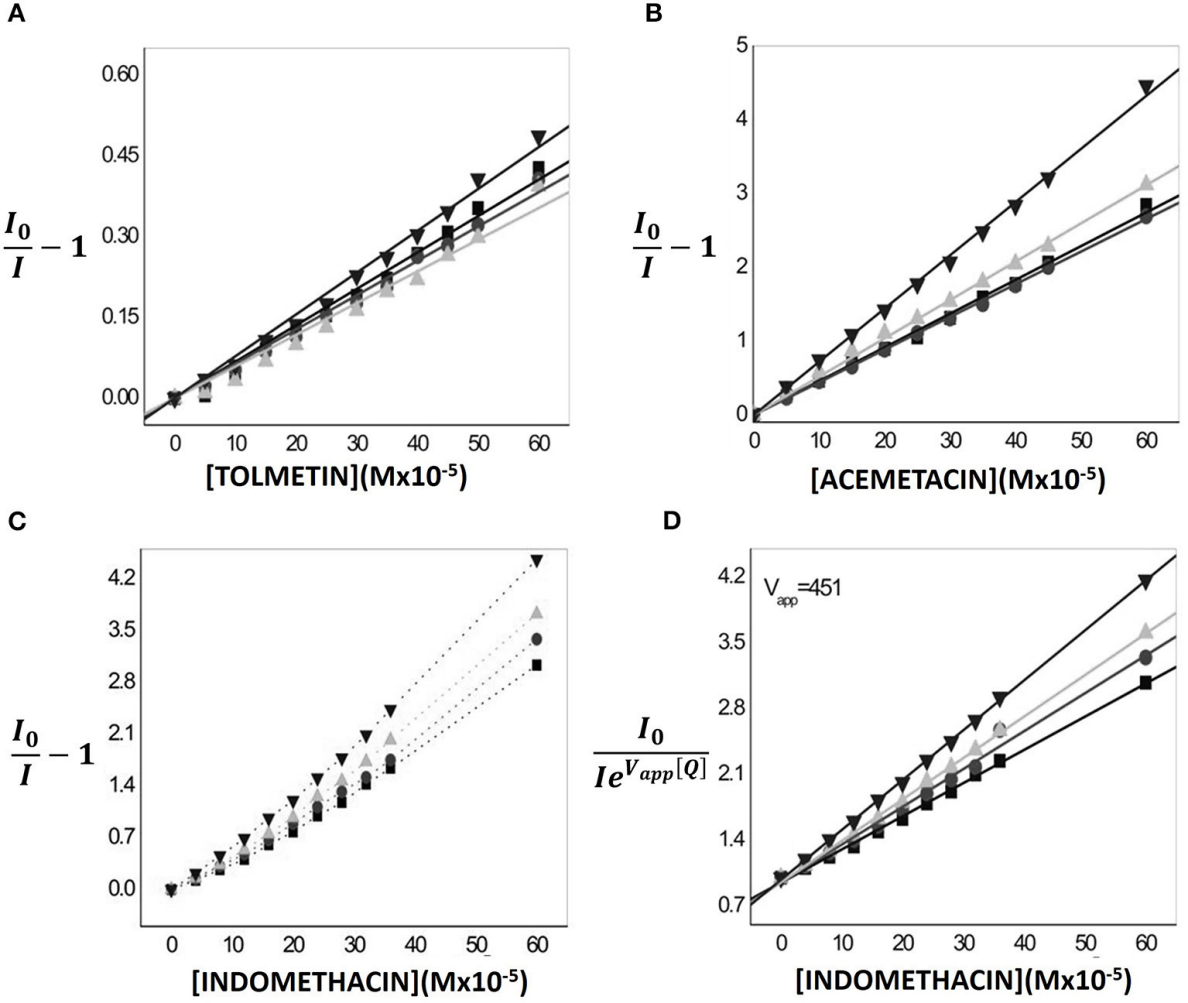
Variations of relative fluorescence intensity (I/I_0_) at 25.0°C of 2-AS (▾), 6-AS (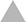) 9-AS (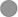) and 12-AS (■) probes incorporated in MLVs (500 μM) as function of increasing concentrations of Tolmetin **(A)** Acemetacin **(B)** and Indomethacin **(C)** in Hepes buffer pH = 7.4 and *I* = 0.1 M. Stern-Volmer linear regressions were also aplied to evaluate the quenching efficiency promoted by Tolmetin **(A)**, Acemetacin **(B)**, and Indomethacin **(D)**.

In the case of Tolmetin and Acemetacin, the fluorescence decreases without meaningful changes in the excitation and emission spectra and only a decrease in band intensity is observed (data not shown), suggesting a fluorescence quenching occurring by collisional processes between drugs and fluorophores (Lakowicz, 2014). This type of fluorescence quenching can be described by the Stern-Volmer linear plots (Equation 5 and Figures 7A,B) where the fluorescence emission intensities of the labeled nanocarrier in the absence (*I*_0_) and in the presence *(I)* of the quencher (drugs) are plotted against the quencher concentration [*Q*] and it is possible to obtain linear relations in which the intercept is zero and the slope corresponds to Stern-Volmer constant, $K_{SV}^{app}$ (Lakowicz, 2014):

(5) $$\begin{array}{r} \frac{I_{0}}{I}-1=K_{SV}^{app}\left[Q\right] \end{array}$$

The efficacy of the quencher (NSAID) to deactivate the fluorescence of each probe can be evaluated by the bimolecular constant, $K_{q}^{app}$ (Equation 6).

(6) $$\begin{array}{r} K_{q}^{app}=\frac{K_{SV}^{app}}{\tau_{0}} \end{array}$$

$K_{q}^{app}$is independent of intrinsic microenvironment changes sensed by each probe, that are reflected in the different values of τ_0_, i.e., lifetime of the excited state characteristic of each probe (Chalpin and Kleinfeld, 1983). However, this parameter is dependent on several other factors: variations in the collisional frequency between probe and quencher; fluorescence quenching efficiency of each probe/quencher pair; radius of probe and quencher and diffusion coefficient, which varies inversely with viscosity of the medium. Considering that the fluorophore and quencher radius, as well as, the intrinsic properties of the fluorophore (τ_0_ and diffusion coefficient) are constant values for all probes, then $K_{q}^{app}$ variations reflect changes in the collisional frequency between fluorophore and quencher. These variations, which are dependent on the relative proximity between probe and quencher may be used to infer the relative location of a drug (quencher) in the lipid nanocarrier (Sikaris and Sawyer, 1982; Chalpin and Kleinfeld, 1983).

Table 2 presents the constants $K_{q}^{app}$ obtained for the three NSAIDs studied and for the four labeled lipid nanocarriers.

**Table 2:** Fluorescence apparent bimolecular constants, $K_{q}^{app}$ determined by fluorescence quenching of *n*-AS probes (incorporated in MLVs at 25.0°C) by each NSAID studied.

**Drug**	**2-AS**	**6-AS**	**9-AS**	**12-AS**
	$K_{q}^{app§}$	$K_{q}^{app§}$	$K_{q}^{app§}$	$K_{q}^{app§}$
Tolmetin	0.768 ± 0.001	0.638 ± 0.001	0.545 ± 0.001	0.612 ± 0.001
Acemetacin	5.194 ± 0.006^****^	4.424 ± 0.002^****^	4.804 ± 0.005^****^	5.675 ± 0.005^****^
Indomethacin	4.013 ± 0.006^**^	4.029 ± 0.001^**^	4.086 ± 0.005^**^	4.181 ± 0.002^*^

^§^(x10^−11^) M^−1^s^−1^

*^§^values correspond to the mean and respective standard deviation obtained for all wavelengths of at least three independent assays. Comparisons were performed using a Krauskal-Wallis test followed by Dunn's Post-hoc test, for the following paired observations: $K_{q}^{app}$values obtained for tolmetin vs. $K_{q}^{app}$values obtained for acemetacin and indomethacin*.

**** *P < 0.0001*,

** *P < 0.01*,

* *P < 0.05*.

Regarding Acemetacin, the relative efficiency of fluorescence quenching for the different *n*-AS probes followed the order: 2-AS≈6-AS < 9-AS < 12-AS, indicating that Acemetacin can reach an inner location at the lipid nanocarrier. Tolmetin $K_{q}^{app}$ values are significantly lower than those obtained for Indomethacin and Acemetacin which indicates that Tolmetin interacts with all probes, but to a lesser extent. Furthermore, $K_{q}^{app}$ values suggest a preferential location of Tolmetin at the polar headgroup region of the lipid nanocarriers, since the relative efficiency of quenching followed the order: 2-AS > 6-AS≈9-AS≈12-AS.

The Stern-Volmer plots of the fluorescence quenching provoked by increasing concentrations of Indomethacin are not linear and present positive deviations (Figure 7C). Positive deviations from the Stern-Volmer equation are often observed when the extent of quenching is large and these deviations from linearity indicate that the quenching mechanism cannot be explained solely by a collisional mechanism (Lakowicz, 2014). There is an apparent static quenching component caused by the close proximity of deactivating drug and fluorophore at excitation moment. The quenching is still collisional, but the existence of fluorophores and quenchers located very close to each other causes an immediate fluorescence deactivation and therefore the fluorophore and the drug appear to form non-fluorescent complexes (Lakowicz, 2014). This type of fluorescence quenching is usually interpreted in terms of a “sphere of action” (Equation 7) within which the possibility of deactivation is equal to unity (Lakowicz, 2014).

(7) $$\begin{array}{r} \frac{I_{0}}{I}=\left(1+K_{SV}^{app}\left[Q\right]\right)e^{\left[Q\right]V_{app}} \end{array}$$

Where *I*_0_ and *I* are, respectively, the corrected fluorescence intensity of the fluorophores in the absence and presence of the drug (quencher), [*Q*] is the drug concentration, $K_{SV}^{app}$corresponds to Stern-Volmer constant relative to fluorescence quenching and *V*_*app*_ is the apparent volume of the sphere of action.

By representing graphically $\frac{I_{0}}{(Ie^{[Q]V_{app}}})$ as a function of *[Q]*, linear relations were obtained in which the slope corresponds to $K_{SV}^{app}$ (Figure 7D). Thus, Indomethacin quenching efficiency followed approximately the same order as that obtained for Acemetacin: 2-AS < 6-AS < 9-AS < 12-AS. Increasing values of $K_{q}^{app}$follows the depth of the fluorophore suggesting that, like Acemetacin, Indomethacin is also able to reach a more inner location within the lipid nanocarrier.

#### Studies to predict how drug encapsulation affects the biophysical properties of lipid nanocarrier

To understand the extent to which the lipid nanocarrier system can be affected by the drug loaded, it is important to analyse certain biophysical properties of the nanocarrier system and how they were impacted by the presence of the drug. Indeed, the evaluation and prediction of possible changes in the nanosystem's microviscosity is an important parameter to consider when choosing a lipid nanocarrier. For example, if including the drug in the lipid nanocarrier alters considerably the microviscosity of the system, it can result in the system becoming too fluid to carry the drug during the required time after administration, and the lipid composition should perhaps be revised to convey more rigid domains. Microviscosity changes induced by the NSAID drugs Tolmetin, Indomethacin and Acemetacin were evaluated by measuring the steady-state fluorescence anisotropy of *n*-AS probes incorporated in MLVs made of EPC.

Small changes in the stiffness of the environment surrounding the probe cause changes in the probe's rotational motion (Lakowicz, 2014). Thus, any correlation time variation, θ will reflect a modification of the lipid nanocarrier microviscosity, $\bar{\eta }$ (Equation 8).

(8) $$\begin{array}{r} \theta =\frac{\bar{\eta }\;V}{R\;T} \end{array}$$

Where the temperature (*T*) is in Kelvin (*K*), *R* represents the gas constant and *V* is the rotational unit volume.

The rotational correlation time (θ) of a probe inserted in a lipid nanocarrier can be calculated from the steady-state fluorescence anisotropy *r*_*ss*_ measured values using the following equation (Monteiro et al., 2011; Lakowicz, 2014):

(9) $$\begin{array}{r} \theta =\tau \left(\frac{r_{ss}-r_{\infty }}{r_{0}-r_{ss}}\right) \end{array}$$

**Figure 8:**
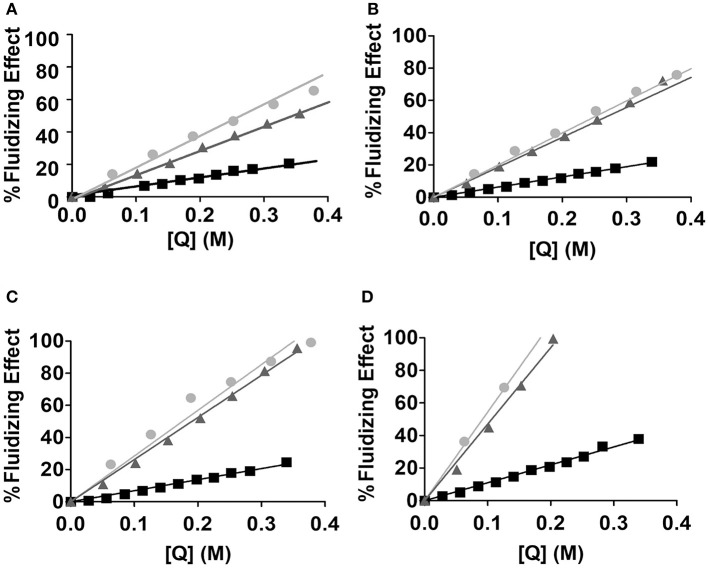
Fluidizing effect (%) caused by increasing concentrations of quenchers [Q]: Tolmetin (■), Acemethacin and (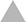) and Indomethacin (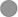), in MLVs of EPC (500 μM, in Hepes buffer pH = 7.4 and *I* = 0.1 M) labeled with *n*-AS probes: **(A)** 2-AS; **(B)** 6-AS; **(C)** 9-AS and **(D)** 12-AS.

Where r_0_ and τ are respectively the fundamental anisotropy of the probe and its lifetime values during the excited state (Vincent et al., 1982), considering that the rotational motion of the probe was not hindered (*r*_∞_ ≈ 0) a condition that is assured when measurements are made above the main phase transition temperature. Since θ gives indications about probe motion, it indirectly reports the fluidity of the lipid environment surrounding the probe and how is it affected by the presence of increasing concentrations of the drug inserted in the lipid nanocarrier. For the three NSAIDs tested (Indomethacin, Acemethacin and Tolmetin) θ values decreased with the addition of increasing drug concentrations indicating a fluidizing effect observable for all the tested drugs (Figure 8).

To establish comparisons between the NSAIDs studied in terms of fluidity efficacy, the respective FE_25_ values were calculated as the concentration in lipid phase (*[Q]* in M) of each compound required to increase the fluidity of the lipid nanocarrier in 25% (Table 3).

**Table 3:** Values of NSAID concentrations (M) required to obtain 25% of fluidizing effect (FE_25_) in the lipid nanocarriers MLVs of EPC (500 μM, pH 7.4) labeled with *n*-AS probes.

**Drug**	**2-AS**	**6-AS**	**9-AS**	**12-AS**
Tolmetin	0.4112 ± 0.0001122	0.3955 ± 0.0001582	0.3633 ± 0.0001453	0.2272 ± 0.00009089
Acemetacin	0.1492 ± 0.0005921^****^	0.1346 ± 0.0005386^*^	0.0879 ± 0.0003514^****^	0.0459 ± 0.0001838^****^
Indomethacin	0.1684 ± 0.0005567^*^	0.1255 ± 0.0005021^****^	0.0953 ± 0.0003812^*^	0.0532 ± 0.0002129^*^

*The values of FE_25_ correspond to the mean and respective standard deviation obtained for at least three independent assays. Comparisons were performed using a Krauskal-Wallis test followed by Dunn's Post-hoc test for the following paired observations: FE_25_ values of Tolmetin vs. FE_25_ values of Acemetacin and Indomethacin*.

**** *P < 0.0001*,

* *P < 0.05*.

### Performance studies

#### Binding to serum albumin to evaluate distribution to the target tissues

After entering the body and being absorbed, drugs reach the systemic circulation where they can strongly bind to plasma proteins. These interactions, can alter their overall characteristics ultimately changing their bioavailability, biodistribution, efficacy, and excretion. In this way, drug-protein interactions and, above all, interactions between drugs and human serum albumin (HSA) should not be overlooked (Trynda-Lemiesz, 2004; Liu et al., 2012). HSA is the most abundant protein in plasma, serving as a storage and transport protein for several endogenous and exogenous compounds. This soluble monomeric protein is produced by the liver and is responsible not only for the transport of lipophilic drugs but also hormones and fatty acids. In addition, HSA promotes osmotic pressure regulation and due to the numerous binding sites identified can reversibly bind to numerous drugs (Liu et al., 2012).

The drug-HSA complex works as a temporary storage, capable of preventing compound elimination and thus, preserves the drug's effective concentration in plasma. This interaction also regulates the free and active blood concentration of the drugs providing a prolonged effect (Carlo and Enrico, 2002). On the other hand, since mostly unbound compounds can permeate cellular membranes, drugs binding to plasma proteins precludes the permeation of those barriers. Therefore, if the drug, which is being used in a therapeutic formulation is extremely bound to plasma proteins and the reversal binding kinetics are slow, only a small amount of drug will be available to permeate membranes and induce the desired physiological effect (Kerns and Di, 2008).

Succinctly, due to HSA high concentration in plasma and drugs binding affinity to HSA, drug-HSA interactions are an important factor to be considered on the development of new drugs and have been broadly studied by various methods (Yang et al., 2014). Bearing this in mind, the affinity to HSA of the NSAID Clonixin and of a formulation DSPC:DODAP(2:1) LUVs loaded with Clonixin was assessed by fluorescence quenching of protein residues, highlighting the intrinsic fluorescence contribution of tryptophan, located in the hydrophobic cavity and, in a smaller amount, tyrosine.

Both performed studies of HSA with the addition of increasing concentrations of Clonixin and HSA with the addition increasing concentrations of DSPC:DODAP(2:1) loaded with Clonixin, have shown a decrease in the intensity of HSA intrinsic fluorescence, indicating a binding between HSA and ligands under study (data not shown). Following a set of specific conditions, a balance between protein free form and bound form can be established. This balance is commonly quantified in terms of the dissociation constant (*K*_*d*_) for the binary complex at equilibrium and can be determined by fluorescence quenching according to Equation 10 (Copeland, 2000; Azevedo et al., 2013):

(10) $$\begin{array}{r} K_{d}=\frac{{\left[HSA\right]}_{free}{\left[ligand\right]}_{free}}{\left[HSA-ligand\right]} \end{array}$$

In this way, *K*_*d*_ can be determined according to a Langmuir isotherm that establishes a relation between the concentration of the complex (*[HSA-ligand]*) and the concentrations of free ligand and HSA (*[HSA]*_*free*_ and *[ligand]*_*free*_):

(11) $$\begin{array}{r} \left[HSA-ligand\right]=\frac{{\left[HSA\right]}_{free}}{1+\frac{Kd}{{\left[ligand\right]}_{free}}} \end{array}$$

The fluorescence quenching of tryptophan and tyrosine residues is equivalent to the concentration of the complex (*[HSA-ligand]*). Thus, by measuring the fluorescence emission of HSA at the wavelength of maximum intensity (λ_*max*_ = 344 *nm*) in the absence of ligands, and considering this value as 100% fluorescence intensity, it is possible to calculate a fluorescence quenching percentage obtained by adding increasing ligand concentrations. Plotting the fluorescence quenching values as function of ligand concentration and applying a non-linear fit according to Equation 12, *K*_*d*_-values can be determined (Copeland, 2000; Azevedo et al., 2013):

(12) $$\begin{array}{r} Fluorescence\;quenching\;\left(\%\right)=\frac{y_{\max}\times n}{1+\frac{K_{d}}{\left[ligand\right]}} \end{array}$$

Where, y_*max*_ is the maximum value of fluorescence quenching reached and *n* are the number of binding sites.

**Figure 9:**
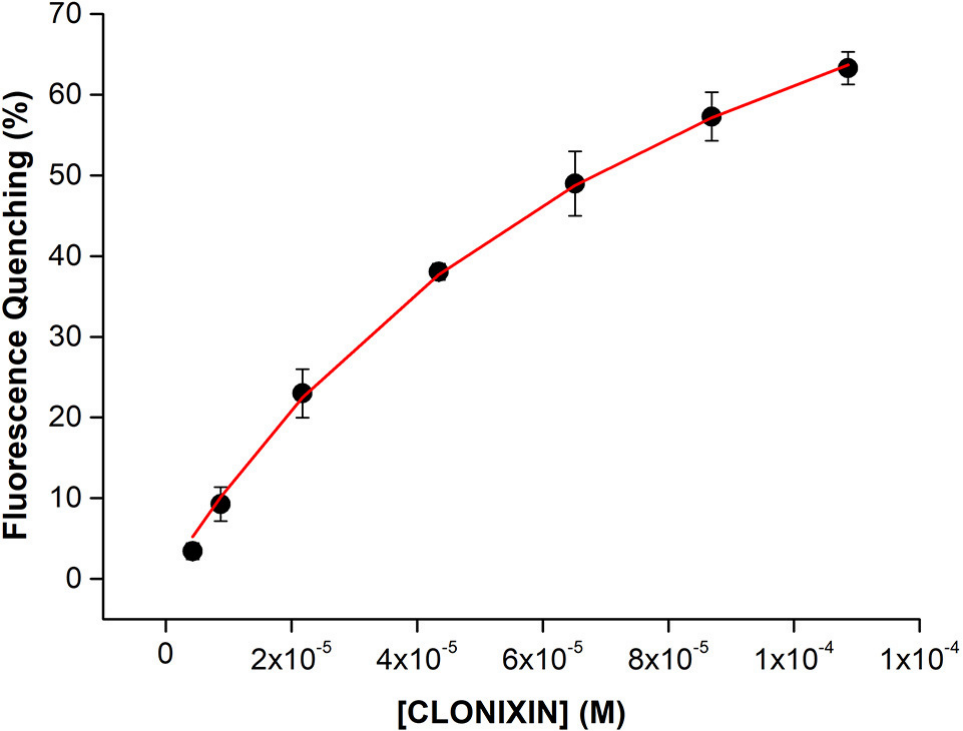
HSA fluorescence quenching (%) as a function of increasing Clonixin concentrations (4.3 × 10^−6^, 8.7 × 10^−6^, 2.2 × 10^−5^, 4.3 × 10^−5^, 6.5 × 10^−5^, 8.7 × 10^−5^, and 1.1 × 10^−4^ M). A non-linear fit according to Equation 12 is presented. Experimental data is presented as average value ± standard deviation of 3 independent assays).

As an example, in Figure 9, it is presented HSA fluorescence quenching (%) due to the complex formation of HSA-Clonixin as function of NSAID concentration (4.3 × 10^−6^, 8.7 × 10^−6^, 2.2 × 10^−5^, 4.3 × 10^−5^, 6.5 × 10^−5^, 8.7 × 10^−5^, and 1.1 × 10^−4^ M) where a non-linear fit of the experimental data was achieved by application of Equation 12.

As can be observed in Figure 9 there is an increase in fluorescence quenching with the increase of Clonixin concentration, proving drug binding to HSA. By applying a non-linear fitting according to Equation 12, it was possible to calculate not only the *K*_*d*_ = 9 × 10^−5^ ± 8.4 × 10^−6^ M but also the number of specific binding sites of HSA (*n* = 1 ± 0.008) involved in the binding of this plasma protein to Clonixin.

The relative affinity or binding strength of protein-ligand complexes (*K*_*b*_) is inversely proportional to the value of *K*_*d*_ meaning that a lower *K*_*d*_ value implies a greater binding strength. Thus, the binding constant and the number of binding location sites results obtained for both interactions, HSA-Clonixin and HSA-DSPC:DODAP(2:1) loaded with 15% of Clonixin, are presented in Table 4.

**Table 4:** Binding constant (K_b_) and number of binding locations (n) of Clonixin and DSPC:DODAP (2:1) loaded with 15% of Clonixin to HSA.

	**K${}_{\text{b}}^{\text{§}}$**	***n***	***R*^2^**
Clonixin	111 ± 10	1 ± 0.008	0.999
DSPC:DODAP (2:1) + 15% Clonixin	143 ± 39^****^	1 ± 0.052^ns^	0.938

*^§^(x10^−2^) M^−1^*.

*The values of K_b_ and n correspond to the mean and respective standard deviation obtained for at least three independent assays. Comparisons were performed using non-parametric Mann-Whitney test, comparing cumulative distributions between Formulation + 15% drug vs. drug*.

**** *P < 0.0001*;

ns *, not significant*.

According to the results presented in Table 4 it is possible to conclude that both studied ligands strongly bind to HSA in only one binding site. However, there is a significant difference between the strengths of these bindings (*P* < 0.0001).

#### Studies of nanocarriers uptake and intracellular drug distribution

To further monitor Daunorubicin's intracellular release dynamics of LUVs of DSPC:DODAP (2:1) a FLIM technique was employed, which is able to distinguish between the “free state” and “DNA-bound state” of the drug by measuring its fluorescence lifetime with high spatial resolution inside of the cell.

In the FLIM system used the fluorescence photon emission decay is stored in histograms. The decay time of each pixel in the FLIM image is generated from the recorded histogram. With this method, we observe the release dynamics of the nanocarrier drug content inside the cell at exact location, evaluating the distribution of the mean lifetime of the drug by a multi-exponential fit of the experimental data.

Daunorubicin, as other anthracycline drugs, presents a mean lifetime larger upon DNA binding in contrast with its free state (Dai et al., 2008; Than Htun, 2009; Chen et al., 2012; Basuki et al., 2013). However, besides the DNA intercalation there is also a propensity of anthracycline drugs of being oxidized to semiquinone, an unstable metabolite, which quickly reacts with oxygen to produce reactive oxygen species (Zhu et al., 2016). This results in drug's decay time decrease, due to quenching by the oxygen radical formed.

The time-resolved fluorescence decay curves of the Daunorubicin in the intracellular environment was evaluated by a multi-exponential model. This multi-exponential analysis was necessary because the lifetime of a fluorescent molecule strongly depends on its surrounding. Moreover, cells are a dynamic and complex system with a slight auto fluorescence. For that reason a large distribution of the decay times is expected, instead of single decay. A bi-exponential model was used and allowed to distinguish two components. One of the components is associated with the drug, whereas the second component has a slow decay time, around 3 ns with a very low amplitude. This component is associated to the cell auto fluorescence.

**Figure 10:**
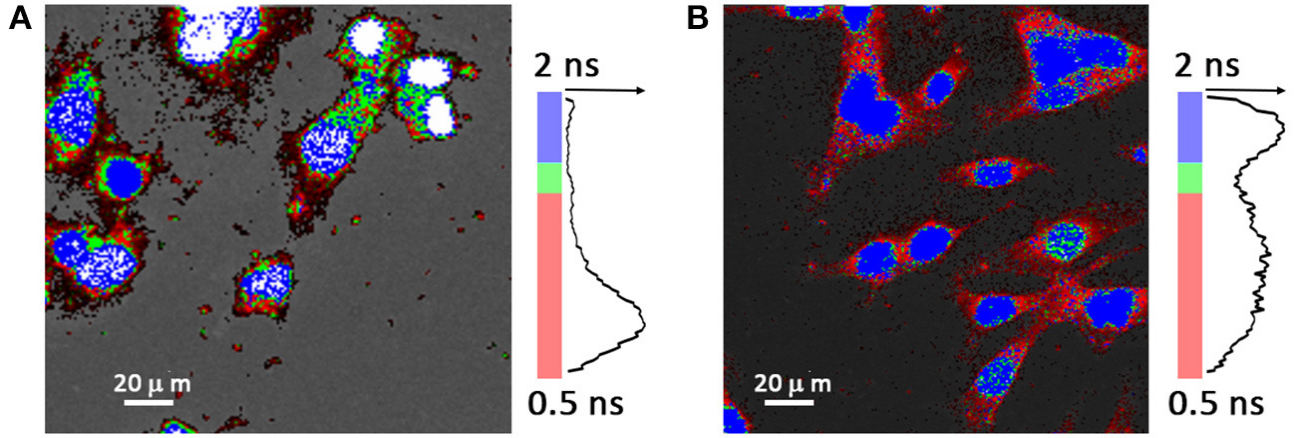
FLIM images showing cell uptake of DSPC:DODAP (2:1) nanocarriers loaded with Daunorubicin (0.5 μM) using HeLa cells after **(A)** 15 min of incubation and **(B)** 4 h of incubation. In the image no membrane dye was employed and a false color range (0.5–2.0 ns) was used according to the fluorescence lifetimes measured. Blue color corresponds to the longer lifetime (~2 ns), while green color corresponds to intermediate lifetime (~1.5 ns) and red color corresponds to shorter lifetimes (~0.5 ns). Fluorescence intensity histogram is represented along side the respective lifetime color bar.

Anthracyclines toxicity mechanism depends on DNA intercalation, to inhibit its transcription. In this regard Figure 10 provides a solid evidence that confirms the successful entrance of the drug at the specific target, nucleus. Figure 10A represents the intracellular distribution profile of the drug after cells being incubated during 15 min with the lipid nanocarrier containing Daunorubicin. It is observed that the major drug content is still in the cytoplasm as some nucleus are still uncolored. In the respective histogram the fast component dominates. However, in the colored picture it is already visible that a small fraction of the drug has already reached the nucleus. Figure 10B represents the drug distribution profile after 4 h of cellular incubation with loaded lipid nanocarriers. In the respective histogram a second band, corresponding to a slow decay, appears and it is accompanied by the increase of the fluorescence intensity in the nucleus. This indicates a drastic increase of the anthracycline drug concentration inside the nucleus and suggests drug intercalation within the DNA. In colored picture presented in Figure 10B it is possible to observe longer lifetime values correspondent to drug-DNA complexes (blue colored nucleus), as well as shorter decay time of 1.5 ns (green colored nucleus) probably due to quenching by the oxygen radical formed and cytoplasm (red colored) where drug is not bound to DNA, thus presenting the shortest decay time.

## Discussion

Comparing lipid aggregates with different organizational complexities, HDPC micelles have the advantage of being more homogeneous, causing less spectral interference, namely lack of light scattering and simpler and faster preparation thus, being an efficient way to predict results of studies with liposomes (Cuccovia et al., 1999). However, in this study, Log*D* values of Naproxen in micelles of HDPC were significantly smaller than Log*D* values of Naproxen in liposomes (Table 1). This might be related with the described high lipophilicity of this NSAID that possesses Log*P* in octanol/aqueous system of 3.96 (Yoo et al., 2003). Indeed, liposomes provide higher distribution opportunities for lipophilic drugs because, in comparison with micelles, liposomes possess lipid bilayers (Bozzuto and Molinari, 2015). On the other hand, concerning the use as lipid nanocarriers, liposomes are considered to be the most popular drug-carrier system known to date (Bozzuto and Molinari, 2015). LUVs, presenting a single bilayer and a fairly large volume of aqueous solution in the interior, are preferable for more hydrophilic drugs, whereas MLVs with a higher entrapment volume and greater number of lipid bilayers are preferred for more lipophilic ones (Pinheiro et al., 2011). Also the release kinetics is considered different for these types of vesicles, and LUVs are known for having faster drug release rates (Bozzuto and Molinari, 2015). Despite Naproxen's high lipophilicity the drug presented no significant difference regarding its distribution in MLVs or LUVs. In fact the equal affinity of Naproxen for either liposomal structures may be related to the presence of methyl groups and a naphthalene based planar molecular structure allowing an efficient interpenetration within the vesicle lipids by adopting a molecular conformation more adaptable to the phospholipid chains.

Fluorescence spectroscopy has proved to be a more suitable method than derivative UV-Vis spectrophotometry for drug Log*D* determination in liposomes/aqueous systems. Log*D* assessment by UV-Vis spectrophotometry was difficult due to the strong light scattering spectral interference of the lipid vesicles at low wavelength values. However, even though similar results of Log*D* of Naproxen were obtained in MLV either by UV-Vis derivative spectrophotometry or by fluorescence spectroscopy, fluorescence emission bands have lower light scattering effects, which are further eliminated with the first derivative of the spectra. Fluorescence spectroscopy also enabled the Naproxen Log*D* calculation in LUVs, which was not possible by UV-Vis derivative spectroscopy due to the strong spectral interference of the lipid vesicles.

The high distribution of Naproxen in micelles, LUVs and MLVs confirmed by the current experimental results suggest an efficient encapsulation of this NSAID in lipid nanocarriers, a strategy that could be of interest to improve drug pharmacokinetics, namely its bioavailability after an oral administration (Marjan et al., 2017).

**Figure 11:**
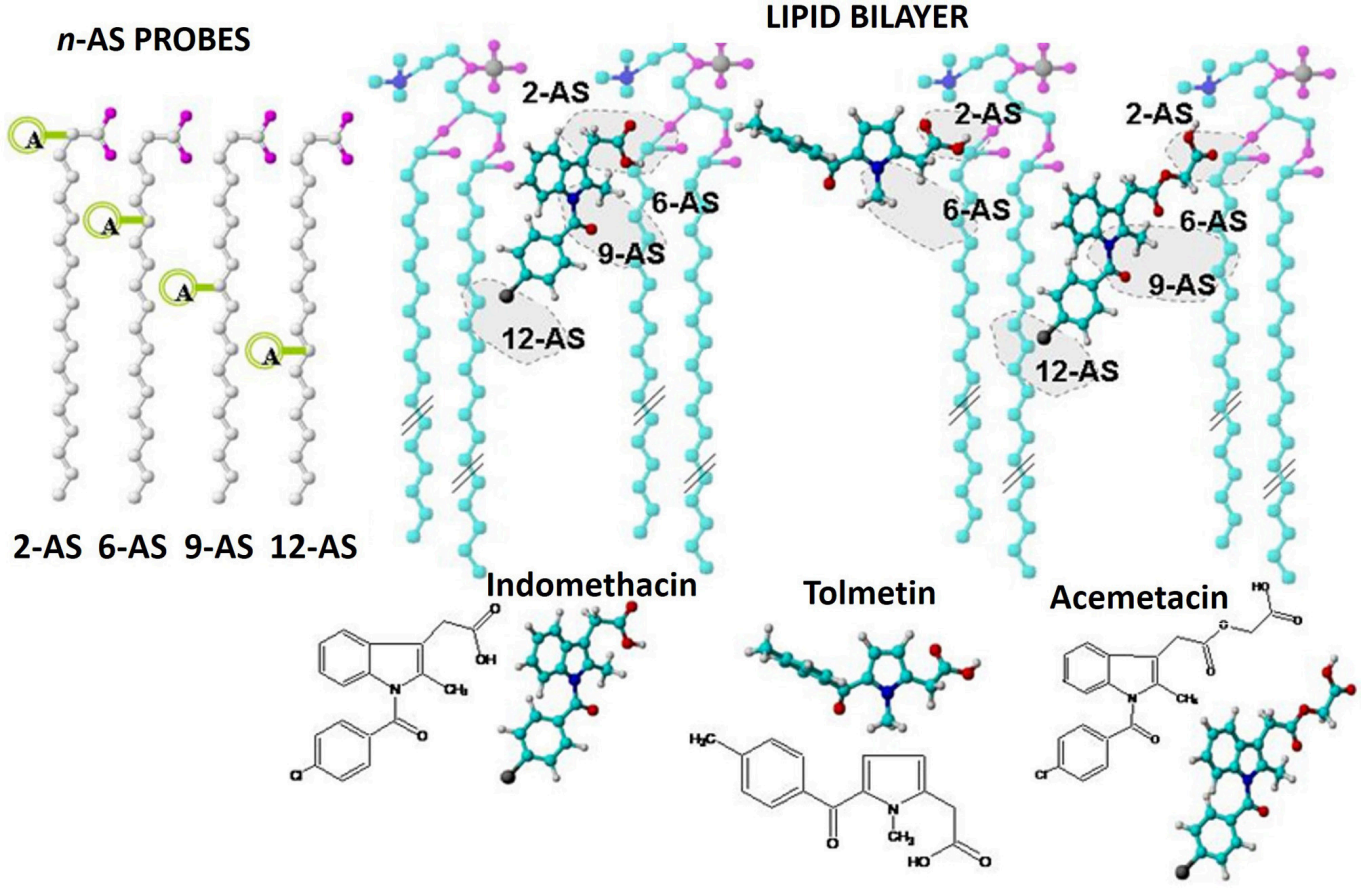
Schematic representation of the possible location/orientation of Tolmetin, Acemetacin, and Indomethacin molecules within the lipid nanocarrier indicating the chemical groups likely responsible for fluorescence quenching of *n*-AS probes.

Besides predicting drug distribution in lipid nanocarriers, it is possible to foresee the drug location or molecule orientation within the phospholipid components of a nanocarrier system. A schematic representation of drugs possible location and molecular orientation within the lipid nanocarrier (MLVs of EPC) is presented in Figure 11.

In the present study, Acemetacin was able to quench the fluorescence of all the probes probably due to an orientation of the molecule anchored by its carboxyl group to the polar nanocarrier headgroups and with its halogen, which constitutes a strong deactivating group, situated at the deepest level of the lipid nanocarrier near the 12-AS probe. Regarding the location of Indomethacin within the lipid nanocarrier, the sphere of action model was used to define the quenching mechanism of the *n*-As probes by the drug, resulting in a prediction of the localization similar to that obtained for Acemetacin. Considering the significantly lower $K_{q}^{app}$values determined for Tolmetin (Table 2), it is expected that the penetration of this drug at lipid bilayer level will be significantly reduced. The same type of conclusions was obtained for the same NSAIDs when their location was predicted in LUVs of EPC (Lucio et al., 2004) or in LUVs of other lipid components (DMPC) with other membrane probe (DPH) acting as molecular ruler (Lúcio et al., 2009). This indicates that the drug location by fluorescence quenching is a robust method to predict drug location and orientation within a lipid nanocarrier. However, the $K_{q}^{app}$ obtained in the previous works (LUVs) were significantly smaller than the $K_{q}^{app}$ obtained in this work (MLVs), perhaps because by possessing a greater number of lipid bilayers MLVs have a higher drug distribution within those bilayers and thus the quenching efficiency is enhanced.

Ultimately, predicting drug location and orientation within a lipid nanocarrier is very important during the formulation development, since it can provide information about the nanocarrier optimization. For instance, if similar to Tolmetin, a drug is located at the surface of the nanocarrier, its release maybe very fast and the protection conferred by the nanocarrier might be reduced during the drug delivery. In such cases the developing team might consider changing the nanocarrier composition to allow a deeper drug penetration, or even establish stronger interactions between the drug and the nanocarrier surface by grafting ligands to the drug that can avoid its rapid release.

After analyzing the drug distribution and the drug location/orientation within the lipid nanocarrier it is also essential to understand the impact of drug loading in the biophysical properties of the nanocarrier (Peetla et al., 2009). Indeed, a drug that is highly distributed within the nanocarrier, may affect differently the nanocarrier microviscosity according to the drug orientation and location. It has been described that lipid assemblies have a microviscosity gradient, being more ordered at the polar headgroup regions and less ordered at the acyl fatty acid chains regions (Davis, 1983; Siminovitch et al., 1983; Seelig and Seelig, 2009). Moreover, studying the impact of drugs in the nanocarrier biophysical properties is paramount as a great reduction of microviscosity can turn the nanocarrier into a very leaky system that is not able to fulfill the role of drug carrier with a controlled drug release (Peetla et al., 2009).

In the current study we have reached the conclusion that all NSAIDs tested fluidized the membrane in a concentration-dependent manner, after which an index was established to compare the effectiveness of membrane fluidization (FE_25_). The lower FE_25_ value, the lower concentration of drug required to have a 25% fluidizing effect on the lipid nanocarrier and therefore the efficacy of drug to induce membrane fluidity is greater. Thus, by the analysis of the determined FE_25_, it is possible to establish the following order of fluidization efficacy of the lipid nanocarriers (MLVs of EPC) by the NSAIDs studied: Indomethacin>Acemetacin>Tolmetin. A similar conclusion was obtained for the same NSAIDs when their location was predicted in LUVs of EPC (Lucio et al., 2004). The FE_25_ values calculated herein are also useful for aiding in the optimization of the nanocarrier loaded with the drug. In fact concentrations of drug loaded in the lipid nanocarrier that induce a fluidizing effect over 25% should be avoided at the risk of turning the nanocarrier into a very permeable system. In this regard FE_25_ values are a good indicator of the maximum amount of drug that should be loaded in the nanocarrier system.

In addition to analyzing the differences observed between the ability of the studied NSAIDs to fluidize the membrane as a whole, it is also important to compare the results obtained for each probe, since *n*-AS probes are located within the lipid nanocarrier at different depths and can therefore provide information about the fluidity gradient in these regions. All the NSAIDs studied increase membrane fluidity for all probes and the observed order is: 2-AS < 6-AS < 9-AS < 12-AS. Our results are in agreement with the literature, since according to lipid membranes fluidity gradient, probes 2, 6 and 9-AS being located nearer the polar headgroup region, a more ordered region, are less susceptible to perturbations than the probe 12-AS located closest to the center of the lipid bilayers of the lipid nanocarrier system (Davis, 1983; Siminovitch et al., 1983; Seelig and Seelig, 2009). In addition, changes in membrane fluidity by NSAIDs corroborate the findings of the location studies and there is a parallelism between drug location within the lipid nanocarrier and effects on membrane fluidity, since a membrane-disturbing drug will have a greater propensity to disturb the surrounding lipid microenvironment.

Besides being useful for the nanocarrier development and optimization in the pre-formulation steps, *in vitro* studies are also useful to provide a first approach of the NT performance. An expected function of *in vitro* studies for therapeutic performance evaluation is helping researchers to understand the factors that dictate the outcome of NT/drug interactions with living systems, in particular with serum proteins and biological barriers (such as the membranes of the target cell tissues) without the need of laborious and expensive *in vivo* studies. Animal models allow the investigation of biological fate of NT/drugs but should be reserved to final stages of their development. Indeed, animal studies are labor-intensive, expensive and controversial. Due to limitations of methodologies and to differences between human/animal pathophysiology it is estimated that only 20% of drug candidates tested successfully in animals passed clinical trials (Perrin, 2014). Moreover, preserving animal health is in agreement with the EU principles (Directive 2010/63/EU) of Replacement (of animals for other relevant *in vitro* models), Reduction (of animals tests) and Refinement (the “Three Rs”). In this regard we propose two *in vitro* studies for a first evaluation of the therapeutic performance of the NT. The first study involves evaluation of the interaction between drug or nanocarrier loaded with drug and the most abundant serum carrier protein–HSA. The second study involves the evaluation of NT or drug cellular uptake, by cells representative of the drug target tissues.

By the results obtained in performance studies where the binding strength to HSA of free Clonixin and DSPC:DODAP (2:1) loaded with Clonixin was compared, it is possible to conclude that under both conditions there is a strong binding to HSA at one binding site. As previously mentioned (Table 4), there is a significant increase in the binding strength when the drug is carried in the lipid nanosystem, stressing the need to make improvements in the developed formulation. Indeed positively charged nanocarriers have higher affinity to negatively charged HSA residues. To reduce HSA binding and improve drug circulation time, PEGylation of the nanosystems can be a solution once polyethylene glycol (PEG) addition to the nanocarrier surface is commonly used to: avoid recognition by the RES cells, reduce the opsonization and plasma protein binding and consequently prolong the systemic circulatory life improving the desired physiological effect (Shi et al., 2002).

Finally the cellular uptake of lipid nanocarriers loaded with Daunorubicin was investigated in cancer cells using FLIM. This *in vitro* assay is also extremely useful to evaluate the therapeutic performance of a nanocarrier loaded with drug, since it is possible to follow drug distribution inside the cells (in the case of a fluorescent drug) or even the nanocarrier distribution if a labeled nanocarrier is applied. In our study, two incubation times of cancer cells and NT loaded with anticancer drug Daunorubicin were tested to evaluate the performance of the NT regarding the capacity for an intracellular deliver of the drug. From the images obtained it was possible to conclude that even at short incubation times (15 min) there was a distribution of the drug in the cytoplasm, whereas for longer incubation periods (4 h) the drug has visibly reached the cellular nucleus where it will exert its effect. Therefore, the nanocarrier system appears to be very effective delivering the drug in comparison to other nanocarrier systems used for anthracycline drugs that reported longer incubation periods for an effective drug distribution (Dai et al., 2008; Basuki et al., 2013).

## Conclusion

We present a toolbox of biophysical studies as strategies to analyze:

- Drug distribution coefficient (Log*D*) between the lipid phases of the nanocarrier and the aqueous phase by derivative spectroscopy, leading to the determination of drug encapsulation. As an a lipophilic drug, Naproxen, was analyzed, and the LogD values >3.0 confirmed the lipophilic nature of the drug and the adequacy of lipid nanocarriers for its encapsulation
- Drug location in lipid nanocarriers by fluorescence quenching where the decrease on fluorescence intensity of extrinsic probes was measured to indicate the drug distribution and possible orientation within the nanocarrier. Acemetacin and Indomethacin can reach an inner location at the lipid nanocarrier while being anchored with its carboxylic moiety at the polar headgroup. The least observed quenching effect suggested that Tolmetin is probably located at the polar headgroup region of the lipid nanocarriers and this superficial location may translate in a fast drug release from the nanocarriers.
- Drug effect on NT biophysical properties using fluorescence anisotropy. This study is important because a great increase of the fluidity of the nanocarrier lipid system can turn it into a too leaky and not so stable carrier for the drug. Fluorescent anisotropy measurements indicated that the drugs deeply buried within the lipid nanocarrier (Acemetacin and Indomethacin) where the ones that had a greater fluidizing effect which can also translate in a faster drug release.
- NT interaction with plasma proteins by measuring fluorescence quenching of intrinsic probes (protein fluorescent residues), as this is paramount to assure that the NT will reach the target tissues and will not have an exceeding circulation time. Clonixin after encapsulation in a lipid nanocarrier DSPC:DODAP (2:1) has still a strong binding to serum albumin, at one binding site, suggesting the need to produce a stealth nanosystem.
- Cellular uptake of NT analyzed by FLIM. As an example we have followed the uptake of lipid nanocarriers loaded with Daunorubicin by cancer cells. From the images obtained it was possible to conclude that at short incubation times (15 min) there was a distribution of the drug in the cytoplasm, whereas for longer incubation periods (4 h) the drug has reached the nucleus.

Results obtained provide an insight at the use of spectroscopic techniques as a toolkit for *in vitro* screening of drug-membrane biophysical interactions to predict at an early stage of development *in vivo* aspects related with lipid nanotherapeutics performance.

## Author contributions

EF and TS performed all experiments and data analysis except FLIM measurements. HG performed FLIM measurements and analysis. ML planned and supervised all experiments and was responsible for writing the manuscript.

### Conflict of interest statement

The authors declare that the research was conducted in the absence of any commercial or financial relationships that could be construed as a potential conflict of interest.
